# Machine learning improves online self-referral for inflammatory rheumatic diseases: a registry-based validation study

**DOI:** 10.1007/s00296-026-06220-2

**Published:** 2026-06-30

**Authors:** Jonathan Bamberger, Sebastian Kuhn, Cay-Benedict von der Decken, Stefan Kleinert, Johannes Knitza

**Affiliations:** 1 Institute for Digital Medicine, School of Medicine, Philipps-Universität Marburg, Baldingerstrasse 1, 35043 Marburg, Germany; 2 RheumaDatenRhePort (RHADAR), Erlangen, Germany; 3 Medizinisches Versorgungszentrum Stolberg, Stolberg, Germany; 4 Klinik für Internistische Rheumatologie, Rhein-Maas Klinikum, Würselen, Germany; 5 Praxisgemeinschaft Rheumatologie Nephrologie Erlangen, Rheumatologische Schwerpunktpraxis, Erlangen, Germany; 6 Rheumatologie/Klinische Immunologie, Universitätsklinikum Würzburg, Medizinische Klinik und Poliklinik 2, Würzburg, Germany

**Keywords:** Rheumatic diseases, Machine learning, Surveys and questionnaires, Referral and consultation, Triage, Decision support systems, Clinical

## Abstract

Referral delays and errors in inflammatory rheumatic diseases (IRDs) are common. RhePort is a German online self-referral tool that estimates IRD probability based on a structured questionnaire and expert-derived algorithm. This study evaluated whether machine learning could improve IRD discrimination of RhePort. In this retrospective internal validation study, 1,333 unique RhePort questionnaires linked to rheumatologist-confirmed diagnoses were analyzed. A shared stratified five-fold cross-validation design was used for Logistic Regression, Neural Network, XGBoost, and LightGBM models. For each algorithm, feature selection and Bayesian hyperparameter optimization were performed, followed by evaluation of ensemble strategies. Discrimination, calibration, clinically relevant operating points, and SHAP-based interpretability were assessed against the original RhePort score. IRD prevalence was 35.6% (475/1,333). LightGBM achieved the strongest individual performance, with an area under the receiver operating characteristic curve of 0.791 using 30 features. A weighted ensemble of LightGBM and Logistic Regression further improved performance to 0.815 and achieved the lowest Brier score (0.166). At 95% sensitivity, specificity increased from 9% for RhePort to 32%; at 90% sensitivity, specificity increased from 15% to 48%. The most influential predictors included C-reactive protein, painful small joints, dactylitis pattern, sex, and erythrocyte sedimentation rate. Ensemble AUC-ROC was 0.828 among 746 patients with CRP and/or ESR information and 0.786 among 587 patients without these laboratory data. Machine learning improved IRD classification over the current expert-derived RhePort score and identified questionnaire items that may be omitted without loss of performance, demonstrating the potential of machine learning to enhance rheumatology referral.

## Introduction

Inflammatory rheumatic diseases (IRDs) are chronic immune-mediated conditions affecting approximately 3% of the population [1]. Early diagnosis and initiation of treatment are crucial to reduce irreversible damage, radiographic progression, and disability [2]. Despite this importance, substantial delays between symptom onset and rheumatology consultation persist. In Germany, a multicenter study reported a median delay of approximately 30 weeks from musculoskeletal symptom onset to rheumatology consultation [3]. However, registry-based care data indicate that the time to diagnosis and care pathways vary substantially between rheumatic disease entities [4]. Similar patterns have been reported internationally [5–7].

These delays arise from non-specific early symptoms, difficulty distinguishing inflammatory from non-inflammatory conditions in primary care, and inefficiencies in referral pathways [8–10]. Only 25–40% of patients referred to rheumatologists are ultimately diagnosed with an IRD, leading to inefficient use of limited specialist resources and prolonged diagnostic delay [11–13]. Several non-digital approaches have been evaluated to improve rheumatology referral, with mixed success [14].

The European Alliance of Associations for Rheumatology (EULAR) explicitly recommends considering digital pre-assessment of patients to improve the referral process [15], particularly in light of the growing potential of digital technologies in rheumatology [16]. RhePort is a German online self-referral tool that collects patient-reported symptoms and inflammatory markers through a structured questionnaire and estimates the probability of inflammatory rheumatic disease using an expert-derived rule-based scoring algorithm [17–19]. Based on this score, patients are automatically assigned different levels of referral urgency, with higher scores corresponding to faster access to rheumatology appointments [18, 19]. While RhePort enables earlier access to specialist care for some patients, its diagnostic performance remains limited, with only moderate discrimination and low specificity, resulting in high rates of false-positive referrals [18–20]. Nevertheless, the cut-off used in daily practice was associated with an approximately doubled probability of IRD and higher IRD rates among physician-referred patients [19]. Similar limitations have also been reported for other digital triage tools in rheumatology [21, 22]. Machine learning (ML) offers a promising approach to improve questionnaire-based triage. Previous work showed that basic ML models can substantially outperform the previously used version of the RhePort score, increasing discrimination and specificity while maintaining high sensitivity [18]. However, prior studies applied ML at a simpler methodological level, evaluated an earlier RhePort version, cohort and did not evaluate the combination of LightGBM-based boosting, systematic feature selection, and ensemble techniques. This study therefore aimed to assess whether advanced ML models can enhance the identification of inflammatory rheumatic diseases and to identify key discriminatory features.

## Methods

### RhePort questionnaire

RhePort is a rheumatology-specific online self-referral and appointment triage platform developed within the RHADAR network to prioritize patients with musculoskeletal complaints according to their probability of an inflammatory rheumatic disease (IRD) [18, 19]. The questionnaire version used in the present study was RhePort 1.3, a fixed 23-question instrument with several subquestions covering patient characteristics, musculoskeletal symptoms, systemic features, and laboratory parameters including C-reactive protein (CRP), erythrocyte sedimentation rate (ESR), rheumatoid factor (RF), anti-CCP antibodies, and HLA-B27 [19]. As in earlier RhePort versions, each question contributes a predefined percentage weight and each answer is linked to a specific factor, resulting in an expert-derived weighted sum score intended to reflect IRD probability and urgency of rheumatologic evaluation [18, 19]. Compared with earlier versions, RhePort 1.3 additionally incorporated extended serological input and rule-based elements to improve recognition of rheumatoid arthritis and axial spondyloarthritis [19]. For the present machine learning analysis, the raw questionnaire responses rather than the final RhePort score were used as model inputs; after restructuring the exported questionnaire data, these responses corresponded to 37 analyzable question groups. In clinical use, patients with a score below 1.0 are considered unlikely to have an IRD, whereas a score above 1.0 is used as the operational threshold leading to an appointment for rheumatologic evaluation [19]. Higher scores are intended to reflect greater urgency, and the prospective RhePort 1.3 study additionally evaluated the higher score ranges of > 2.4 and > 4.0 [19]. At the operational cut-off of > 1, RhePort 1.3 was associated with an approximately twofold higher IRD risk, 73% sensitivity, and 42% specificity [19].

### Data source, cohort definition, and outcome

This retrospective prediction study used RhePort questionnaires linked to specialist-confirmed diagnoses in the RheumaDatenRhePort (RHADAR) registry [17]. The source analysis file contained RhePort submissions completed between March 11, 2021 and November 16, 2025. Records were eligible when a questionnaire could be linked to a rheumatologist-confirmed diagnosis and a unique RhePort identifier. If multiple questionnaires were available for the same patient, only the most recent submission was retained, yielding a final cohort of 1,333 unique patients. The Ethics Committee of Philipps-University Marburg confirmed that formal ethical approval was not required for this anonymous study (reference: 26–187 ANZ). The diagnostic composition of the final cohort is summarized in Table 1. The primary prediction target was the presence of an inflammatory rheumatic disease at final rheumatologist-confirmed assessment. Final diagnoses were grouped into IRD and non-IRD categories, resulting in 475 IRD cases (35.6%) and 858 non-IRD cases (64.4%). Reporting was adapted based on recommendations for the design, conduct, and reporting of survey studies [23].

**Table 1:** Diagnostic categories in the final analysis cohort. Percentages are reported relative to the full study population (*n* = 1,333)

Diagnostic category	*n* (%)
Inflammatory rheumatic disease	475 (35.6)
Rheumatoid arthritis	226 (17.0)
Psoriatic arthritis	69 (5.2)
Polymyalgia rheumatica	61 (4.6)
Axial spondyloarthritis	36 (2.7)
Undifferentiated arthritis	21 (1.6)
Crystal arthropathies	21 (1.6)
Reactive arthritis	7 (0.5)
Other connective tissue diseases	7 (0.5)
Sjögren syndrome	7 (0.5)
Systemic lupus erythematosus	6 (0.5)
Systemic sclerosis	5 (0.4)
Giant cell arteritis / temporal arteritis	5 (0.4)
ANCA-associated vasculitis	3 (0.2)
Autoinflammatory syndromes	1 (0.1)
Non-inflammatory rheumatic disease	858 (64.4)

#### Feature engineering and model development

The original RhePort questionnaire responses were transformed from 37 question groups into 156 model features. Ordered variables were encoded ordinally, single-choice categorical items were one-hot encoded, multi-select symptom fields were decomposed into binary indicators, and continuous laboratory and joint-count variables were harmonized numerically. Four classification approaches were evaluated: regularized Logistic Regression, a multi-layer perceptron neural network, XGBoost, and LightGBM. For each algorithm, backward feature elimination started from the full 156-feature set and iteratively removed the least informative variables. The three feature subsets with the highest cross-validated discrimination were then tuned with Bayesian hyperparameter optimization using 100 Optuna trials per subset, yielding 12 final base models in total. Four ensemble strategies were subsequently applied to the out-of-fold predictions of all 12 models: simple averaging, greedy forward selection (iteratively adding models to maximize AUC-ROC), stacked generalization (logistic regression meta-learner with inner five-fold cross-validation), and a weighted variant of the greedy ensemble in which optimal blending weights were determined via Nelder-Mead optimization. Missing continuous predictor values within included records were handled by median imputation inside the relevant model pipelines.

## Validation design, baseline comparison, and reproducibility

All analyses used a single shared stratified five-fold cross-validation split generated once with shuffling and a fixed random seed of 42, then persisted and reused across all pipelines. Out-of-fold predictions were retained for every patient and served as the common basis for model comparison, ensemble construction, targeted threshold analyses, and interpretability analyses. No separate internal hold-out set was created because the study prioritized directly comparable internal estimates across all candidate models in a moderate-sized cohort. The original RhePort score was evaluated on the same patient cohort as the primary baseline comparator. Discrimination was assessed primarily with AUC-ROC. Threshold-dependent operating characteristics were summarized with sensitivity, specificity, positive predictive value, and negative predictive value, using the F1-optimal threshold for the main comparison tables. Clinical screening utility was additionally examined at operating points targeting 70%, 90%, and 95% sensitivity. Calibration was assessed with the Brier score. Model interpretability was assessed using Shapley additive explanations (SHAP), which quantify the contribution of individual features to model predictions and were aggregated to evaluate the importance of original RhePort questionnaire items. In each fold, the fitted estimator was trained on four folds and evaluated on the held-out fifth fold, so each patient received one out-of-fold prediction from an estimator that had not been fitted on that patient. No external cohort was available. Feature elimination, hyperparameter selection, ensemble selection, and performance reporting were not embedded in a fully nested outer cross-validation loop. The existing ensemble was additionally evaluated in patients with versus without CRP and/or ESR information using the overall F1-selected threshold. Core analyses were implemented in Python 3.11.5 using scikit-learn 1.6.1, LightGBM 4.6.0, XGBoost 3.0.2, Optuna 4.7.0, SHAP 0.46.0, NumPy 1.26.4, Pandas 2.2.3, SciPy 1.15.1, and Matplotlib 3.10.0.

## Results

### Patient dataset

The final study cohort comprised 1,333 RhePort questionnaires with verified diagnoses. Table 1 summarizes the final diagnostic categories assigned by the treating rheumatologists. Of the 1,333 patients, 475 (35.6%) were diagnosed with an inflammatory rheumatic disease, with rheumatoid arthritis representing the most common IRD diagnosis (226/475, 47.6%). The majority of patients were female (59.7%) and younger than 61 years of age (73.3%). Only a minority reported symptom duration shorter than 6 weeks (6.8%). Most patients had previously seen a general practitioner (71.6%). Laboratory information on CRP and/or ESR was available for 746/1,333 patients (56.0%). Fatigue was reported by 966/1,333 patients (72.5%), and the most common comorbidities were osteoarthritis (33.6%) and obesity (29.9%). Baseline characteristics stratified by outcome are shown in Table 2. Compared with the non-IRD group, the IRD group contained a larger proportion of men (53.7% vs. 32.9%), patients aged 61 years or older (35.4% vs. 19.9%), and patients with CRP and/or ESR information (61.9% vs. 52.7%).

**Table 2:** Baseline demographic and clinical characteristics stratified by IRD status

Characteristic	Overall *n* = 1,333	IRD *n* = 475	Non-IRD *n* = 858
Female sex	796 (59.7)	220 (46.3)	576 (67.1)
Male sex	537 (40.3)	255 (53.7)	282 (32.9)
Age < = 45 years	474 (35.6)	143 (30.1)	331 (38.6)
Age 46–60 years	503 (37.7)	156 (32.8)	347 (40.4)
Age > = 61 years	339 (25.4)	168 (35.4)	171 (19.9)
Age missing	17 (1.3)	8 (1.7)	9 (1.0)
Symptoms < = 6 weeks	90 (6.8)	47 (9.9)	43 (5.0)
Symptoms 6 weeks-3 months	210 (15.8)	101 (21.3)	109 (12.7)
Symptoms 3–6 months	245 (18.4)	102 (21.5)	143 (16.7)
Symptoms 6–12 months	198 (14.9)	54 (11.4)	144 (16.8)
Symptoms > 1 year	328 (24.6)	95 (20.0)	233 (27.2)
Symptom duration missing	262 (19.7)	76 (16.0)	186 (21.7)
Prior GP visit	954 (71.6)	334 (70.3)	620 (72.3)
CRP and/or ESR available	746 (56.0)	294 (61.9)	452 (52.7)
Fatigue	966 (72.5)	330 (69.5)	636 (74.1)
Osteoarthritis	448 (33.6)	140 (29.5)	308 (35.9)
Obesity	398 (29.9)	131 (27.6)	267 (31.1)

### Model performance and clinical operating points

**Fig. 1:**
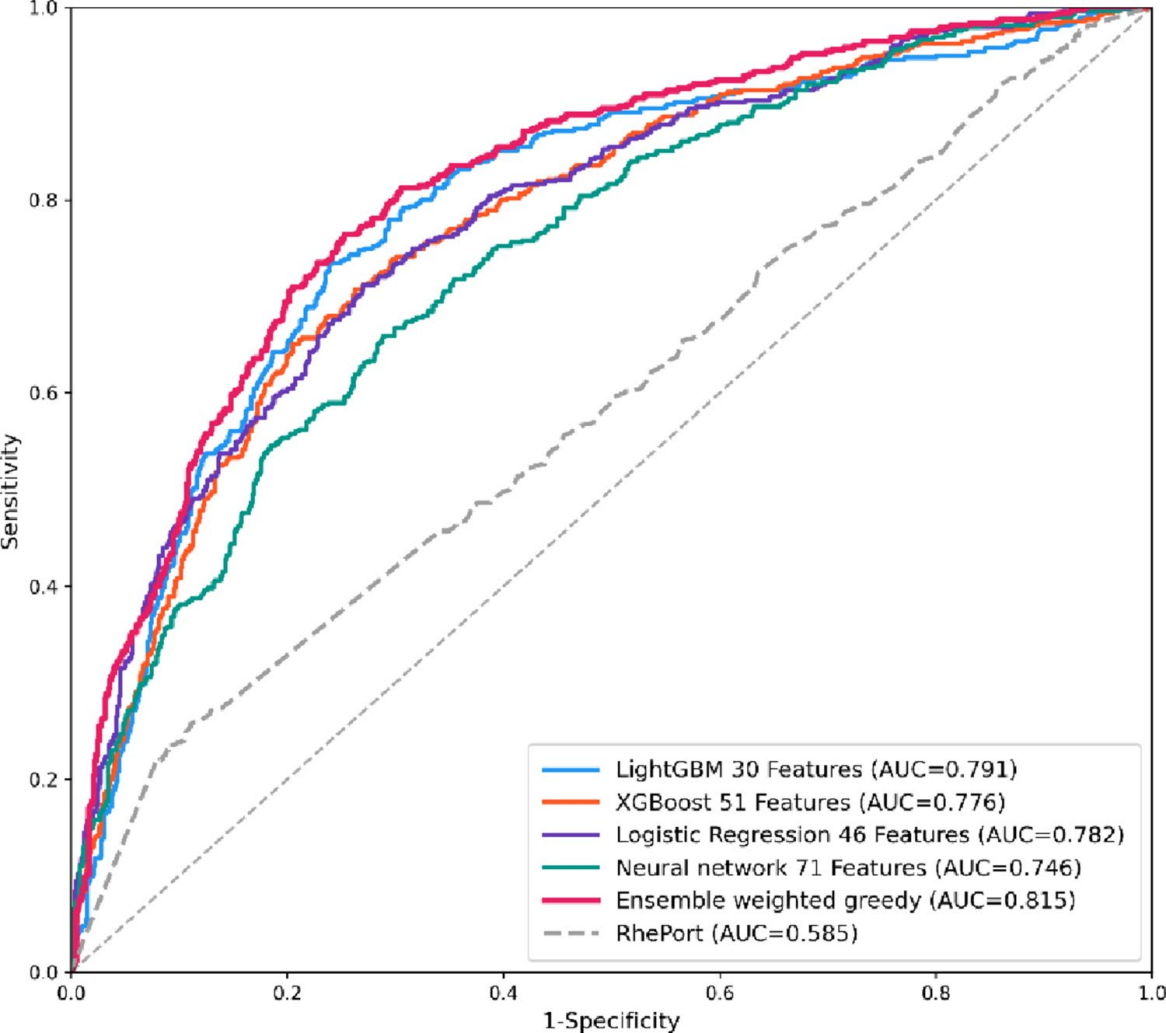
ROC comparison of the weighted greedy ensemble, the best individual machine learning models, and the RhePort score. The ensemble achieved the highest overall discrimination

All machine learning models outperformed the current RhePort score (AUC-ROC 0.585; Table 3). LightGBM was the best individual model with an AUC-ROC of 0.791 using 30 features, followed by Logistic Regression (0.782), XGBoost (0.776), and the neural network (0.746). The weighted greedy ensemble achieved the highest overall discrimination, with an AUC-ROC of 0.815, and Fig. 1 summarizes the corresponding ROC curves.

**Table 3:** Compact comparison of the best-performing models and the RhePort score. Sensitivity and specificity are reported at the F1-optimal operating threshold used in the source analysis. Lower Brier scores indicate better calibration

Model	Features	AUC-ROC	Sensitivity	Specificity	Brier
Weighted greedy ensemble	77	**0.815**	0.764	0.745	**0.166**
LightGBM	**30**	0.791	0.733	**0.760**	0.176
Logistic Regression	46	0.782	0.749	0.679	0.177
XGBoost	51	0.776	0.712	0.728	0.180
Neural network	71	0.746	0.752	0.606	0.192
RhePort score	n/a	0.585	**0.962**	0.076	0.249

**Fig. 2:**
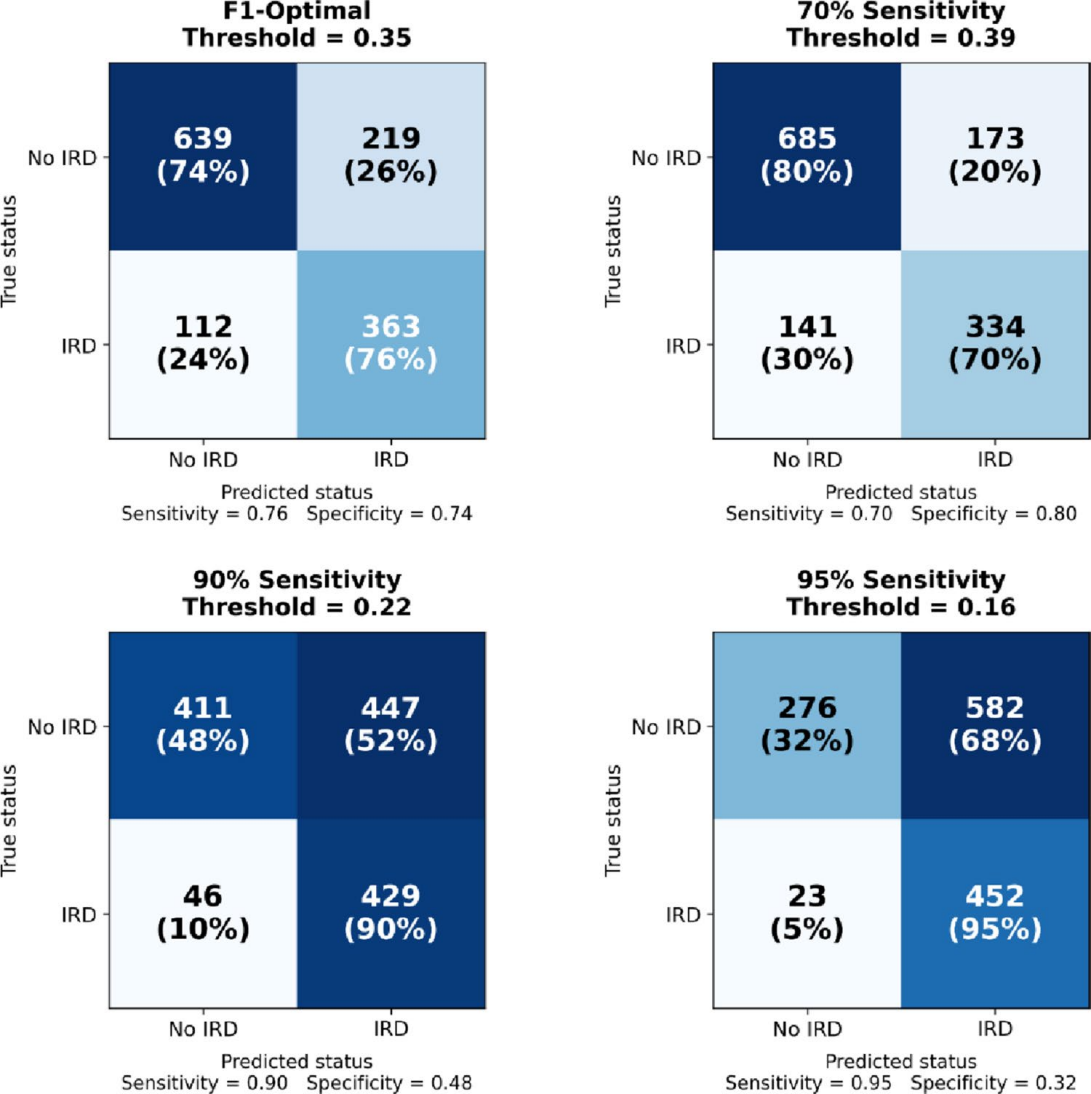
Confusion matrices for the weighted greedy ensemble at the F1-optimal threshold and at clinically relevant operating points targeting 70%, 90%, and 95% sensitivity. Class labels distinguish IRD from No IRD

The final weighted greedy ensemble combined LightGBM and Logistic Regression, with weights of 55.4% and 44.6%, respectively. Relative to the best individual model, this increased AUC-ROC by 3.0%; relative to the current RhePort score, the improvement was 39%. Calibration remained favorable, with the lowest Brier score in the main comparison (0.166). At clinically relevant high-sensitivity thresholds, the ensemble retained substantially greater specificity than the rule-based baseline. At 95% sensitivity, the ensemble reached 32% specificity compared with 9% for RhePort, while negative predictive value increased from 77% to 92%. At 90% sensitivity, specificity was 48% for the ensemble versus 15% for RhePort. The corresponding confusion matrices across selected operating points are shown in Fig. 2. At the 95% sensitivity operating point, the ensemble produced 23 false negatives and 582 false positives, compared with 23 and 779 for RhePort. At the 90% sensitivity operating point, the corresponding counts were 43 and 455 for the ensemble versus 44 and 733 for RhePort. The remaining false-positive burden is therefore still substantial (Table 4).

**Table 4:** Targeted sensitivity comparison between the weighted greedy ensemble and the RhePort score

Target sensitivity (%)	Weighted Greedy			RhePort score		
	Spec. (%)	PPV (%)	NPV (%)	Spec. (%)	PPV (%)	NPV (%)
95	**32**	**44**	**92**	9	37	77
90	**48**	**49**	**90**	15	37	74
70	**80**	**66**	**83**	37	38	70

### Performance by laboratory-data availability

CRP and/or ESR information was available in 746 patients (56.0%), including 294 IRD cases. Using the overall F1-selected ensemble threshold of 0.35, performance was stronger in patients with laboratory data (AUC-ROC 0.828, sensitivity 0.820, specificity 0.712) than in those without laboratory data (AUC-ROC 0.786, sensitivity 0.674, specificity 0.781; Table 5). This subgroup analysis evaluates the existing model under observed missingness and is not a separately retrained laboratory-free model.

**Table 5:** Ensemble performance according to CRP and/or ESR availability at the overall F1-selected threshold (0.35)

Subgroup	*n*	IRD (%)	AUC-ROC	Sensitivity	Specificity	Brier
Laboratory data available	746	39.4	0.828	0.820	0.712	0.167
No laboratory data	587	30.8	0.786	0.674	0.781	0.166

### Interpretability and questionnaire reduction

**Fig. 3:**
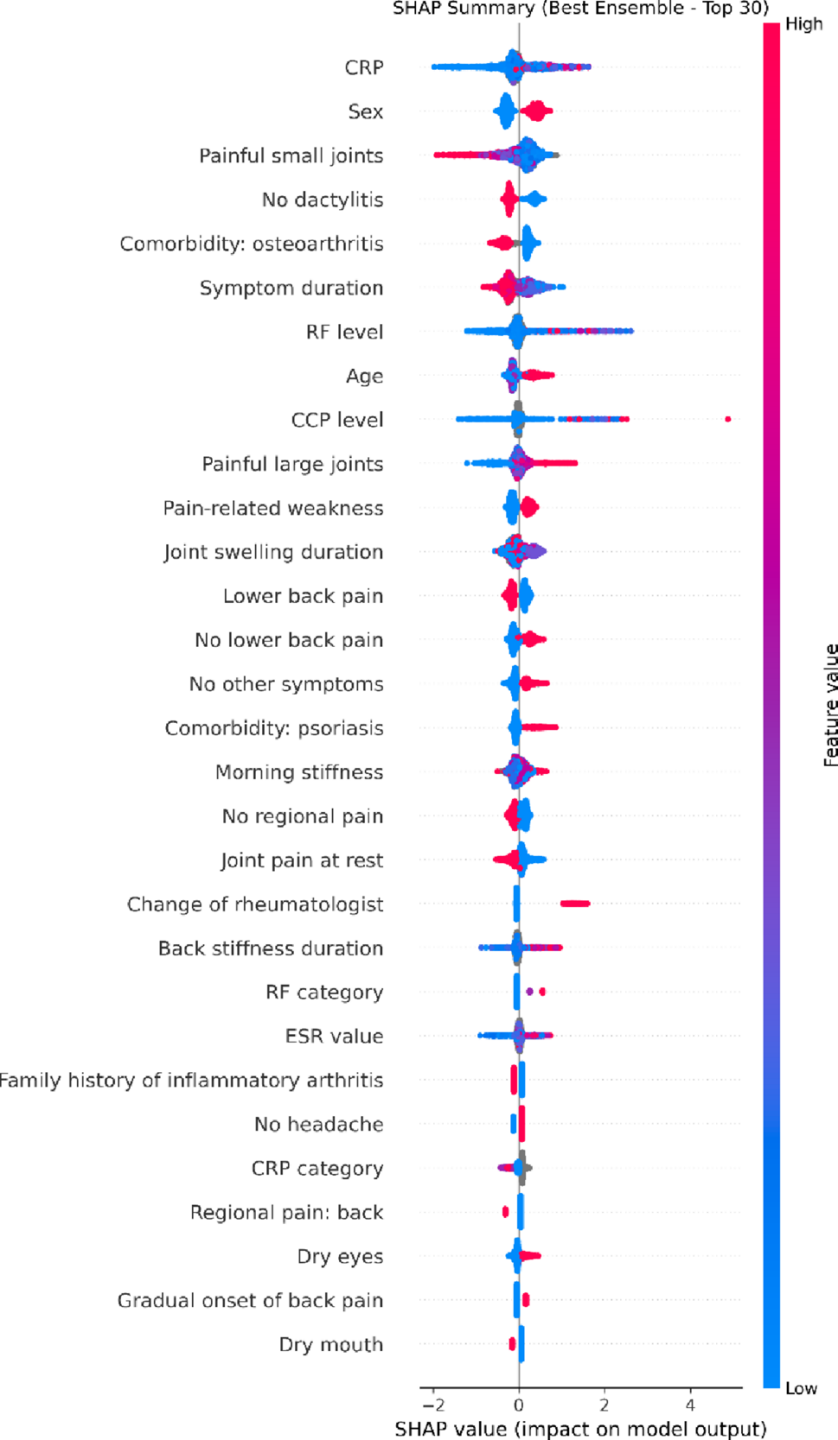
SHAP summary plot for the most influential encoded features. In the binary Sex feature, red points indicate male patients and blue points indicate female patients

**Fig. 4:**
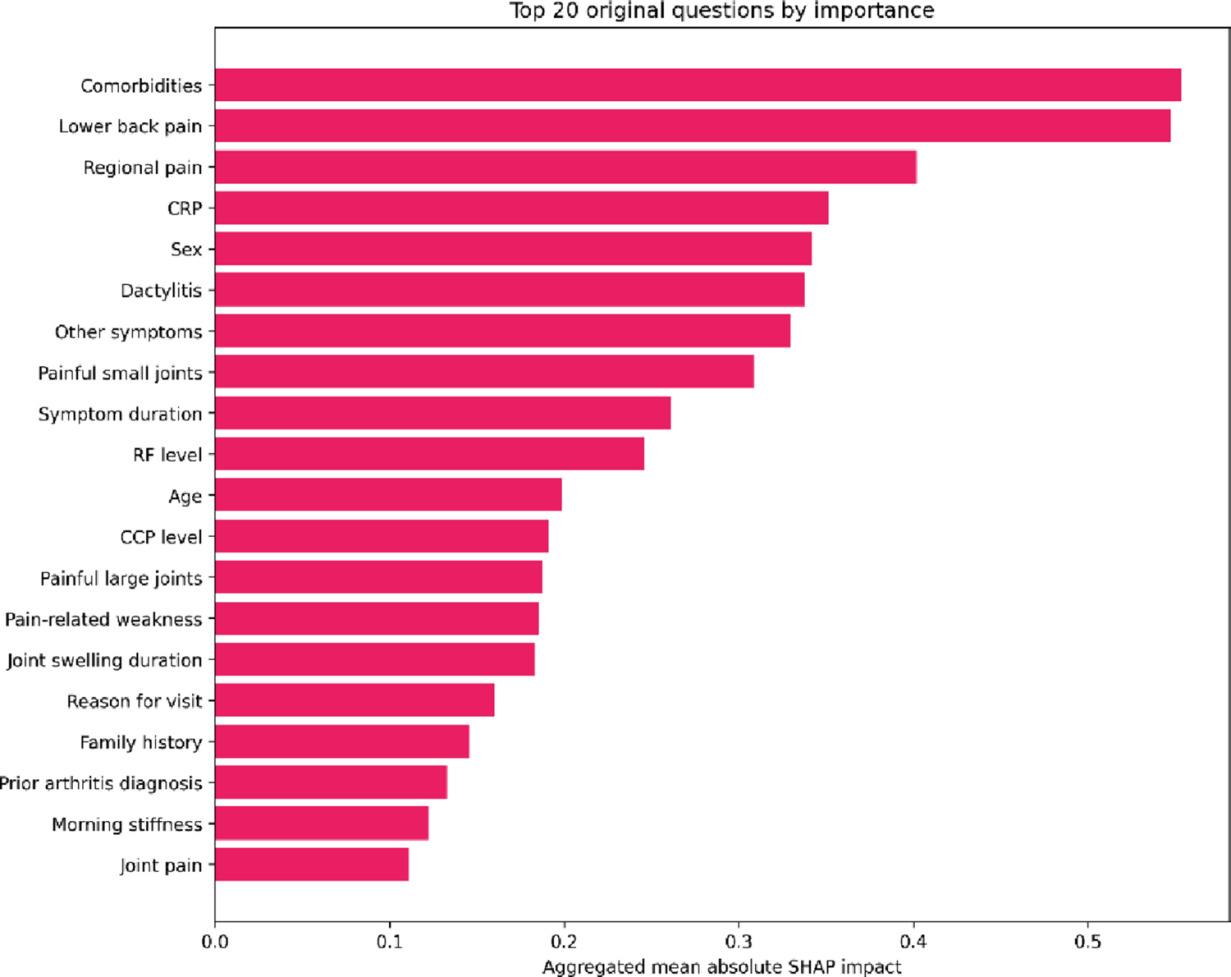
Aggregated SHAP importance at the original RhePort questionnaire-item level

Interpretability analysis showed that the ensemble relied primarily on inflammatory markers and joint-pattern features. The four most influential predictors were CRP (C-reactive protein), the number of painful small joints, dactylitis pattern, and ESR (Erythrocyte sedimentation rate). The reduction findings were not limited to the ensemble. The best individual model, LightGBM, reached its highest AUC-ROC with only 30 features, indicating that strong performance did not require the full encoded feature set. Aggregation from encoded features back to original questionnaire items further showed that only 33 of the 37 question categories contributed at least one useful signal to the final model. Four categories made no measurable contribution: Trauma/Injury, muscle weakness, infection history, and CCP antibody ratio. Figures 3 and 4 summarize the most influential encoded features and the aggregated contribution of the original questionnaire sections.

## Discussion

This study demonstrated that machine learning can improve patient questionnaire-based IRD identification. The diagnostic performance of the final weighted greedy ensemble was 39% better than the current RhePort algorithm. Importantly, this model also exceeded the previously reported best RhePort machine learning result (AUC-ROC 0.737) by 10.6% [18]. The key finding is not only a higher AUC-ROC, but a more useful screening profile: the weighted greedy ensemble preserved high sensitivity while providing substantially better specificity than the current score. These operating points show that the machine learning model maintained a screening-oriented profile while reducing unnecessary referrals much more effectively than the current score. Furthermore, the study identified the most important referral features and questionnaire items that may potentially be omitted. Because all estimates were derived internally and model selection was not fully nested within the validation procedure, the observed magnitude of improvement may be overestimated and should not be interpreted as evidence of generalizable performance in external settings.

### Comparison with previous results

Several methodological differences likely account for the improvement over the AUC-ROC of 0.737 reported by Knitza et al. [18]. The evaluated cohorts had different sizes (1,333 vs. 2,265 patients) with different IRD prevalences (35.6% vs. 30.5%). The inclusion of LightGBM proved important, achieving the highest individual AUC-ROC (0.791) with only 30 features and contributing 55.4% of the ensemble weight. The moderate class imbalance was addressed through stratified cross-validation without resampling, differing from the SMOTE-based approach of the earlier study, as SMOTE can introduce artificial distributional shifts in moderate-sized clinical datasets. The ensemble also compares favorably with other diagnostic approaches. In a prospective multicenter trial, Knitza et al. [20] reported that the former version of RhePort achieved 62% sensitivity and 47% specificity, while the general-purpose symptom checker Ada reached 66% sensitivity and 54% specificity in its top-5 output. These cross-study comparisons are descriptive and should not be interpreted as a direct performance ranking because populations, questionnaire versions, and study designs differed. The clinical relevance is underscored by documented rheumatology workforce shortages in the United States, Germany, and Canada [24–26] and increasing referral delays [3]. False-positive referrals may lead to unnecessary specialist assessment and diagnostic work-up, increasing patient burden and contributing to pressure on limited rheumatology capacity [27]. Conversely, false-negative referrals are clinically more concerning, as delayed specialist assessment may postpone diagnosis and initiation of disease-modifying antirheumatic drugs [18]. EULAR recommendations for early arthritis emphasize rheumatology assessment within 6 weeks of symptom onset and DMARD initiation as early as possible [28]. Therefore, referral algorithms require careful balancing between minimizing missed inflammatory disease and avoiding unnecessary referrals.

### Feature importance and clinical plausibility

SHAP analyses were clinically plausible, with inflammatory markers and joint-pattern variables ranking high, while four questionnaire categories (Trauma/injury, muscle weakness, infection history, CCP antibody ratio) showed no measurable contribution, suggesting future questionnaire simplification. This ranking is broadly consistent with routine inflammatory disease assessment, where acute-phase reactants and peripheral joint involvement carry major diagnostic weight. The prominence of laboratory variables is also notable because these values are optional in the current RhePort workflow. Together, these findings suggest that not all current RhePort questions are required for strong predictive performance and identify concrete candidates for a shorter future questionnaire. For muscle weakness, this finding likely reflects limited discriminatory value in the current cohort rather than absence of symptoms, as muscle weakness was frequently reported but did not contribute measurable signal to the final model. The current analysis identified male sex as a strong IRD predictor. However, this likely reflects bias in the study cohort, in which IRDs were more prevalent among men than women (47.5% vs. 27.6%). Given that IRDs are generally more common in women [29], this finding highlights the need for a larger and more representative training cohort. The number of painful small joints exhibited a non-monotonic effect, with counts above approximately 11 producing negative contributions, indicating that the model distinguishes inflammatory joint involvement from the widespread pain patterns of fibromyalgia or generalized osteoarthritis. Both this study and prior work [18, 19] identified laboratory parameters as top-ranked predictors, consistent with Ehrenstein et al. [30], who reported only 27% diagnostic accuracy for IRD and 19% for RA when rheumatologists were restricted to clinical assessment without prior external workup. The strong contribution of laboratory markers suggests that more consistent availability of laboratory data could further improve performance, as laboratory reporting is currently optional in the RhePort questionnaire. The four apparently non-contributing question categories should therefore be treated as candidates for prospective testing. In the laboratory subgroup analysis, sensitivity at the overall threshold was lower without CRP or ESR information (0.674 vs. 0.820).

### Limitations and future directions

All performance estimates derive from five-fold cross-validation on a single retrospective dataset, which supports internal comparison but does not establish generalizability. The dataset of 1,333 patients remains modest for complex architectures, likely explaining the neural network’s weaker performance (AUC-ROC 0.746). Since laboratory parameters are optional, model performance likely varies with laboratory completeness. The present model should be viewed as a candidate decision-support layer rather than a deployment-ready replacement. Feature selection, hyperparameter optimization, ensemble selection, and final reporting used the same cross-validation framework rather than a fully nested design, which may produce optimistic estimates. The cohort also included only patients who completed RhePort and were linked to specialist diagnoses, creating potential selection, verification, and spectrum bias. Missing laboratory data and changes in case mix, site, time period, or questionnaire version may further limit transportability. Larger training cohorts and external multicenter validation are needed to confirm these promising results. In addition, because the model was developed for binary IRD identification rather than disease-specific diagnosis, its reliability in rare or underrepresented IRD entities remains uncertain. Limited case numbers for conditions such as connective tissue diseases, systemic lupus erythematosus, and vasculitides may constrain subgroup-specific sensitivity and generalizability. Overall the intended role is referral-triage support, not diagnosis or autonomous exclusion of IRD. Implementation should retain clinical oversight and monitor case mix, missing laboratory data, referral volumes, sensitivity, calibration, and subgroup performance. Material calibration drift or sustained loss of sensitivity should trigger reassessment or recalibration.

## Conclusion

These findings highlight the potential of machine learning to improve rheumatology referral. In this study, machine learning substantially improved RhePort IRD identification over the current score and previously reported machine learning approaches. While the model was mainly driven by clinically plausible predictors, feature importance analyses also revealed potentially misleading or confounded features, emphasizing the need for larger and more diverse training cohorts. External multicenter validation is needed to confirm these promising findings.

## Data Availability

The raw data supporting the conclusions of this article will be made available by the authors upon reasonable request.
